# Detailed Correlation between Central Incisor Movement and Alveolar Bone Resorption in Adults with Orthodontic Premolar Extraction Treatment: A Retrospective Cohort CBCT Study

**DOI:** 10.3390/jcm11226872

**Published:** 2022-11-21

**Authors:** Chenghao Zhang, Ling Ji, Zhihe Zhao, Wen Liao

**Affiliations:** 1 State Key Laboratory of Oral Diseases & National Clinical Research Center for Oral Diseases, Department of Orthodontics, West China Hospital of Stomatology, Sichuan University, Chengdu 610041, China; 2 State Key Laboratory of Oral Diseases & National Clinical Research Center for Oral Diseases, West China Hospital of Stomatology, Sichuan University, Chengdu 610041, China

**Keywords:** CBCT, alveolar bone resorption, central incisors, premolar extraction

## Abstract

Background: This study aims to explore the detailed correlation between the movement of maxillary and mandibular central incisors and alveolar bone resorption in adults who had orthodontic premolar extraction treatment. Methods: A total of 63 adult patients (mean age, 24.41 years) who received orthodontic treatment with the extraction of four first premolars were included in this study. CBCT images were obtained before and after treatment. Three-dimensional evaluations of the movement of 252 central incisors (126 maxillary and 126 mandibular incisors) and alveolar bone changes were conducted. Four points were used to describe the incisor movement: C (cusp point), R (root apex point), M (mid-point of root neck), and L (labial cementoenamel junction point). The thickness of labial and palatal alveolar bone was assessed at the crestal, mid-root, and apical levels of incisors. The results were analyzed with Spearman’s correlation and multilinear regression. Results: Matching the measurements of central incisor movement and alveolar bone resorption, significant correlations could be observed. For maxillary central incisors, the labial alveolar bone resorption at the crestal level was correlated with the movement of Point L (r = 0.290, *p* < 0.05), and the labial alveolar bone resorption at the apical level was correlated with Point M (r = 0.387, *p* < 0.05). For mandibular central incisors, the labial alveolar bone resorption at the apical level was correlated with the movement of Point M (r = 0.493, *p* < 0.05) and R (r = 0.498, *p* < 0.01); the palatal alveolar bone resorption at the mid-root level with Point M (r = -0.170, *p* < 0.01); and the palatal alveolar bone resorption at the apical level with Point R (r = 0.177, *p* < 0.01). Conclusions: This study investigated the concrete correlations between central incisor movement and alveolar bone resorption in adults after orthodontic treatment with premolar extraction. It is potentially helpful for orthodontists to have a relatively accurate prediction of alveolar bone resorption based on the specific movements of central incisors and to reduce the risk of alveolar bone resorption by better adjusting the three-dimensional movement types of incisors.

## 1. Introduction

Premolar extraction is a routine orthodontic treatment for correcting severe arch discrepancies, such as severe arch protrusion and crowding. The total extraction frequency of orthodontic treatment is about 25%, and 8.9–13.4% of cases undergo four first premolar extraction (i.e., four first premolars are taken out) [1]. During orthodontic treatment, the closure of the extraction space depends on the mesial movement of the posterior teeth and the distal movement of the anterior teeth [2]. In the process of tooth movement, alveolar bone resorption appears as a major risk [3,4].

Alveolar bone resorption is a potentially adverse outcome following orthodontic treatment [5]. Although most of the alveolar bone resorption is within the clinically acceptable range, a severe alveolar bone reduction would have a harmful impact on the periodontal tissue and cause irreversible damage, including the loss of tooth adhesion, gingival recession, and even tooth loss [6]. A variety of risk factors can cause alveolar bone resorption and affect its severity during treatment, such as age, treatment duration, and tooth position change, among which tooth position change is a factor that could be controlled by orthodontists [7]. 

However, thus far, the correlation between tooth movement and its concomitant alveolar bone resorption remains controversial and not clear enough [8,9,10]. Some previous studies used 2D images (lateral cephalogram or panoramic radiographs) to evaluate the incisor movement and alveolar bone resorption. However, using 2D images to measure 3D objects would lead to unavoidable errors [11]. Compared with 2D images, CBCT provides accurate three-dimensional (3D) performance and thus could improve the reliability and comparability of measurements of dental and skeletal structures in clinical studies [12]. CBCT is now widely applied in dentistry and is especially useful for orthodontic treatment, including clinical diagnosis, treatment planning, avoiding treatment risks, and evaluating prognosis [13]. The more recent studies using CBCT images for the measurements showed that after orthodontic treatment involving the extraction of premolars, the palatal alveolar bone thickness of maxillary central incisors was closely related to the changes in the position and inclination of the incisors [14,15,16,17]. However, due to the small sample size, inconsistent reference lines, and disparate methods of measurement, the level of evidence was relatively low, and thus their results were very different. Moreover, as they did not report the specific type of spatial position movement or the amount of tooth movement, the quality of the studies is not good enough [18]. Therefore, a detailed and comprehensive analysis is still needed to better reveal the correlation between alveolar bone resorption and tooth movement in orthodontic patients treated with premolar extraction.

In clinical research, establishing proper and unified three-dimensional (3D) vectors as a reference standard is a helpful method to describe and understand the structural changes in three dimensions, which could better guide treatment decision making. Our previous study proposed a method that combined a maxilla-based coordinate system and mandibular voxel-based superimposition so that the maxillary and mandibular structural changes could be directly measured and compared with the same 3D vectors [19]. Utilizing this method, in this study, we performed accurate and comprehensive measurements of the movement of maxillary and mandibular central incisors and their associated alveolar bone resorption and, in a detailed and systematic way, constructed their correlation in adult patients undergoing orthodontic treatment with premolar extraction.

## 2. Materials and Methods

### 2.1. Sample Collection

This is a retrospective, cohort study, and patients who received orthodontic treatment with fixed appliances in the Department of Orthodontics, the West China Hospital of Stomatology, Sichuan University (Chengdu, China), from April 2016 to January 2022, were manually filtered using a medical record database of the hospital. The recorded diagnoses and treatment characteristics of patients were browsed. The patients were selected as research samples for this study based on the following inclusion and exclusion criteria:

The inclusion criteria were (1) CBCT images taken within 2 weeks before and after orthodontic treatment; (2) the imaging field of CBCT covering the cranial and maxillofacial skeletal structures from the orbitals to the mandibular body with the imaging data being sufficiently clear and free of artifacts; (3) patients were older than 18 years with all teeth from the central incisors to the second molars and had no supernumerary tooth, tooth defect, or metallic restorations; (4) patients with four first premolars extracted during orthodontic treatment; (5) the use of fixed appliances for orthodontic treatment; (6) moderate anchorage during space closure; (7) healthy periodontal tissue confirmed by both clinical examination and CBCT images, and no pathological alveolar bone resorption; (8) no history of maxillofacial trauma; and (9) complete space closure and good functional occlusion after treatment.

The exclusion criteria were (1) obvious facial asymmetry; (2) the maxillary sinus floor being too low to influence teeth movement; and (3) patients with craniofacial syndrome or systemic disease.

All the CBCT images were taken with the same CBCT machine (3D Accuitomo, Morita Group, Japan), which was set according to the manufacturer’s recommendations (140 × 100 mm FOV, 85 kV, 4.0 mA, and 360° rotation). The voxel size was 125 μm. The CBCT data were then stored in DICOM multifile format.

According to the results of our preliminary experiments and previous research [5], the sample size was calculated using the PASS software (Version 2021; NCSS, LLC; Kaysville, UT, USA; ncss.com/software/pass.). By setting the significance level at 0.05 and power at 0.9, at least 63 samples were needed with an effect size of 0.416. Designed as a before–after comparison, this study needed at least 63 maxillary and mandibular central incisors.

### 2.2. Data Preparation before Measurement

**Figure 1:**
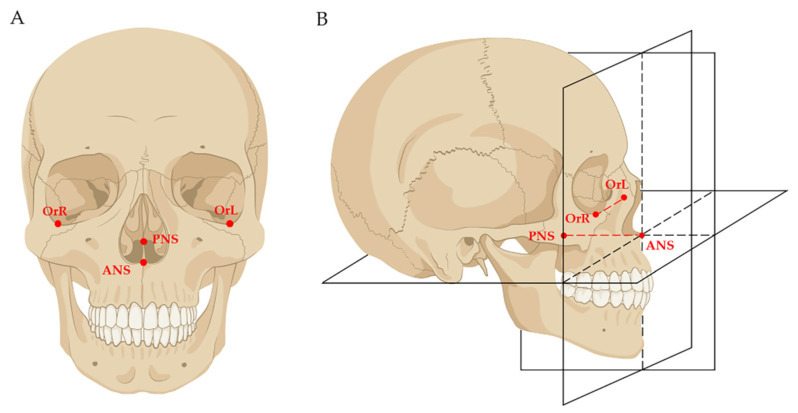
Schematic diagrams of the maxilla-based coordinate system: (**A**) four skeletal landmarks were selected as the basic landmarks for constructing the coordinate system, including ANS (the tip of the anterior nasal spine), PNS (the tip of the anterior nasal spine), OrL (the most inferior point of the left bony orbit), and OrR (the most inferior point of the right bony orbit); (**B**) the horizontal plane was defined as the plane passing through ANS and PNS, while parallel to the OrL–OrR line. The sagittal plane was defined as the plane passing through ANS and PNS while perpendicular to the horizontal plane. The frontal plane was defined as the plane passing through ANS while perpendicular to both the horizontal plane and the sagittal plane.

Before measurement, data were prepared with the method proposed in our past research [19,20]. Firstly, the DICOM data of both pre-treatment (T0) and post-treatment (T1) were imported into the Dolphin software (Version 11.8; Dolphin Imaging and Management Solutions; Chatsworth, CA, USA); mandibular voxel-based superimposition was conducted so that the mandibles of T0 and T1 were superimposed, and the interference of mandibular positional changes caused by orthodontic treatment was eliminated. After that, the data of T1 were reoriented and exported as the T2 data. Secondly, the T0, T1, and T2 data were imported into the Mimics Research software (Version 19.0; Materialise, Leuven, Belgium), and the 3D models were reconstructed. Thirdly, a maxilla-based coordinate system was constructed in T0 and T1 models by using four skeletal landmarks: ANS, PNS, OrL, and OrR (Figure 1). As these four skeletal landmarks in adults were stable, the T0 and T1 coordinate systems were the same. 

**Figure 2:**
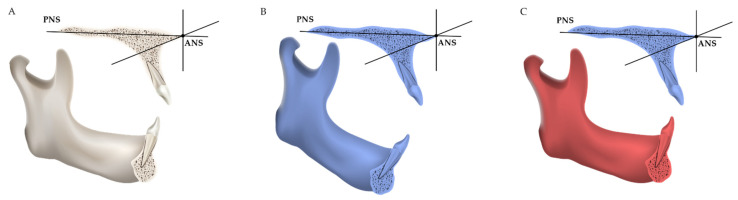
Schematic diagrams of the measurement method: (**A**) T0 structures were in white. T0 coordinate system was used for the measurement of T0 maxillary and mandibular structures; (**B**) T1 structures were in blue. T1 coordinate system could not be used for the measurement of T1 mandibular structures because of mandibular position change; (**C**) T2 (i.e., the reoriented T1) mandibular structures were in red. T1 coordinate system was used for three-dimensional measurement of T1 maxillary and T2 mandibular structures.

Finally, the T0 coordinate system was used for three-dimensional measurement of T0 maxillary and mandibular structures; the T1 coordinate system was used for three-dimensional measurement of T1 maxillary and T2 (i.e., the reoriented T1) mandibular structures (Figure 2).

### 2.3. Measurements of Incisor Movement and Alveolar Bone Resorption

**Figure 3:**
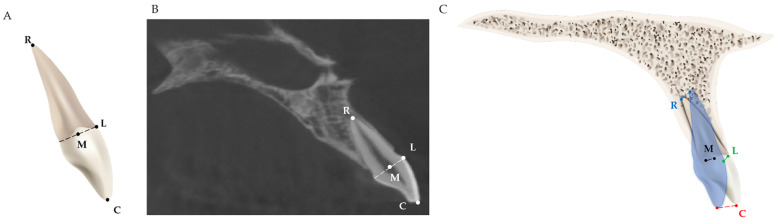
Schematic diagrams of the measurement of incisor movement: (**A**) four dental landmarks were used for measuring the incisor movement; (**B**) dental landmarks located on actual CBCT images; (**C**) the movement of these four landmarks. Red line: the movement of Point C; blue line: the movement of Point R; green line: the movement of Point L; black line: the movement of Point M.

Four dental landmarks on the incisor were used for measuring the three-dimensional movement of the incisor after orthodontic treatment with premolar extraction, as shown in Table 1 and Figure 3.

**Figure 4:**
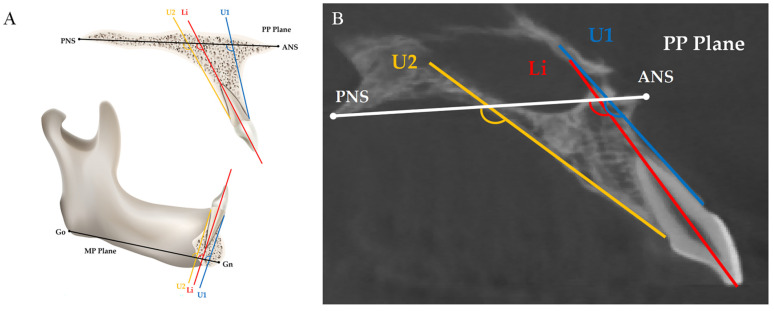
(**A**) Schematic diagrams of the measurement of angular changes in incisors and alveolar ridge. Red line: the long axis of the incisor; blue line: the labial alveolar ridge measurement plane; yellow line: the palatal alveolar ridge measurement plane; (**B**) measurements on actual CBCT images.

Two skeletal basic planes, the long axis of the incisor, and two measurement planes of the alveolar bone were used for evaluating the angular changes in the incisors and the alveolar ridge (Table 2 and Figure 4).

**Figure 5:**
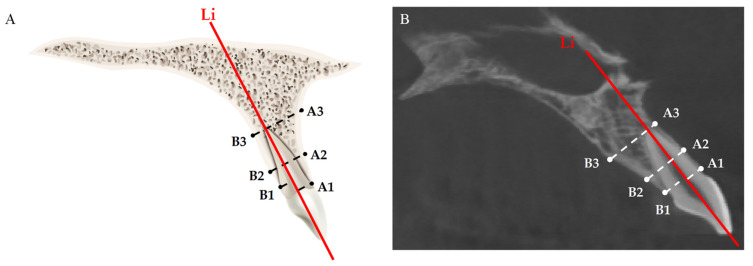
(**A**) Schematic diagram of the measurement of the alveolar bone thickness at crestal, mid-root, and apical levels. A1–Li, labial alveolar bone thickness at crestal level; A2–Li, labial alveolar bone thickness at mid-root level; A3–Li, labial alveolar bone thickness at apical level; B1–Li, palatal alveolar bone thickness at crestal level; B2–Li, palatal alveolar bone thickness at mid-root level; B3–Li, palatal alveolar bone thickness at apical level; (**B**) measurements on actual CBCT images.

Six measurement landmarks on alveolar bone were used for evaluating the changes in alveolar bone thickness at the crestal, mid-root, and apical levels (Table 3 and Figure 5).

### 2.4. Data Analysis and Statistics

Each of the operations and measurements were conducted three times and independently by two operators under identical conditions. The intraclass correlation coefficient (ICC) was used to assess the inter-observer agreement. Statistical analysis was performed with the SPSS software (Version 22.0; IBM, Armonk, NY, USA).

The distribution types of all variables were examined by performing the Kolmogorov–Smirnov test. Normally distributed data (*p* > 0.05) are described by means and standard deviations (x ± s), and non-normally distributed data are described by medians and quartile intervals (M ± Q). The Mann–Whitney U test was used to assess and compare the differences in age and treatment duration between sex groups. Paired *t*-tests were performed to evaluate the incisor movement and changes in the alveolar bone thickness before and after treatment. The threshold of statistical significance was set at 0.05. The associations between the movements of different incisor landmarks and changes in the alveolar bone thickness at different levels were evaluated with Spearman’s correlations. Among these associations, the statistically significant ones were further explored with multiple linear regression analysis. Since conducting multiple analyses on the same dependent variable may result in an increased chance of committing a Type I error, the *p*-value adjusted by Bonferroni’s correction is additionally indicated [25,26].

## 3. Results 

### 3.1. Basic Characteristics of Patients

A total of 252 central incisors from 63 patients were collected for analysis, including 126 maxillary and 126 mandibular central incisors. All 63 patients were treated with maxillary and mandibular bilateral first premolar extraction. Table 4 shows the distribution of the subjects, including their demographic characteristics, treatment duration, etc.

The patients ranged in age from 18 to 42 years, with an average of 24.41 ± 5.80 years. The treatment duration was 31.77 ± 10.30 months. No significant differences were found between the two sexes in terms of age and treatment duration (Table 5).

### 3.2. The Movement of Central Incisors

The movements of the four dental landmarks of both maxillary and mandibular central incisors were, respectively, measured, as shown in Table 6.

### 3.3. Changes in Alveolar Bone

The changes in the alveolar bone thickness at different levels were measured (Table 7). For the maxillary central incisor, the labial alveolar bone at the crestal level (A1–Li, *p* < 0.01), the mid-root level (A2–Li, *p* < 0.001), and the apical level (A3–Li, *p* < 0.05) was significantly absorbed; the palatal alveolar bone at the mid-root level (B2–Li, *p* < 0.01) was absorbed. 

For the mandibular central incisor, the labial alveolar bone was absorbed only at the apical level (A3–Li, *p* < 0.001), and the palatal alveolar bone at both the mid-root level (B2–Li, *p* < 0.001) and the apical level (B3–Li, *p* < 0.001) was significantly absorbed (Table 8). 

**Figure 6:**
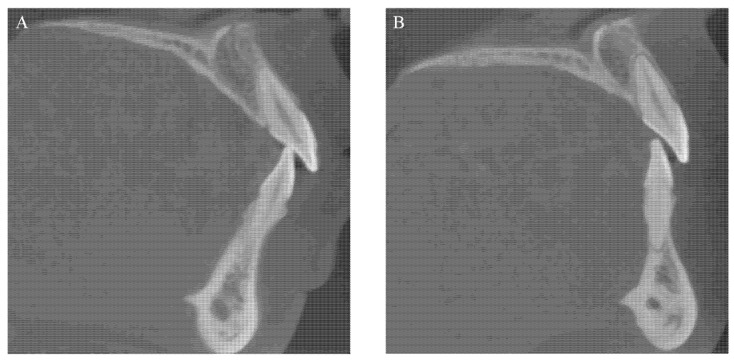
A representative example of alveolar bone resorption in both maxillary and mandibular central incisors after orthodontic treatment: (**A**) alveolar bone status before orthodontic treatment; (**B**) absorbed alveolar bone after orthodontic treatment.

Figure 6 shows a representative example of alveolar bone resorption in both maxillary and mandibular central incisors after orthodontic treatment.

The inclination changes in the central incisor and its associated labial and palatal alveolar ridge were measured (Table 9). No significant angular change during the treatment was found.

The ICC value of the inter-observer agreement for the linear and angular measurements was 0.991 (*p* < 0.001), which indicated the great precision and reproducibility of the measurements between observers.

### 3.4. Factors Related to Alveolar Bone Resorption

To determine the factors correlated with alveolar bone resorption at those levels with significant changes, as shown above, the age, treatment duration, and the movement of the four landmarks of the incisor were analyzed using Spearman’s correlation (Table 10). For the alveolar bone of the maxillary central incisor, the change in A1–Li was correlated with the movement of Point L (r = −0.362, *p* < 0.01); A3–Li with Point C (r = 0.254, *p* < 0.05) and Point M (r = 0.387, *p* < 0.01); and B2–Li with Point C (r = 0.287, *p* < 0.05). However, only one of them attained a *p*-value < 0.0021 (below the Bonferroni cut-off level), emphasizing that the movement of Point M in the maxillary central incisor was positively associated with the labial alveolar bone resorption at the apical level.

For the alveolar bone of the mandibular central incisor (Table 11), the change in A3–Li was correlated with the movement of Point C (r = 0.280, *p* < 0.05), Point R (r = 0.495, *p* < 0.001), Point L (r = 0.349, *p* < 0.01), and Point M (r = 0.485, *p* < 0.001); B2–Li with Point M (r = 0.296, *p* < 0.05); and B3–Li with Point R (r = 0.354, *p* < 0.01). Two of them attained a *p*-value < 0.0028 (below the Bonferroni cut-off level), which indicated that the movement of Point R and Point M were positively associated with the labial alveolar bone resorption at the apical level in the mandibular central incisor.

Among the multiple associations found through the Spearman correlation analysis, the statistically significant ones were further analyzed with multiple linear regression. The results indicated that for the maxillary central incisor, the change in A1–Li was correlated with the movement of Point L (r = 0.290, *p*< 0.05) and A3–Li with Point M (r = 0.387, *p* < 0.05) (Table 12). However, none of them attained a *p*-value < 0.0125 (below the Bonferroni cut-off level).

For the mandibular central incisor, the change in A3–Li was correlated with the movement of Point R (r = 0.498, *p*< 0.01) and Point M (r = 0.493, *p* < 0.05); B2–Li with Point M (r = −0.170, *p* < 0.01); and B3–Li with Point R (r = 0.177, *p* < 0.01) (Table 13). Three of them attained a *p*-value < 0.0083 (below the Bonferroni cut-off level), which indicated that in the mandibular central incisor, the movement of Point R was positively associated with the labial and palatal alveolar bone resorption at the apical level, while the movement of Point M was negatively associated with the palatal alveolar bone resorption at the mid-root level.

## 4. Discussion

Our results reveal the detailed and comprehensive associations between the changes in the spatial position of maxillary and mandibular central incisors and the resorption of the anterior alveolar bones at different levels in adult patients treated with orthodontic premolar extraction. These results were analyzed and established by using Spearman’s correlation and further confirmed by performing multiple linear regression.

The association between tooth movement and alveolar bone resorption in maxillary central incisors is different from that in mandibular central incisors. Their association in the mandibular central incisor showed regularity according to the Spearman correlation analysis: the movement of the incisor point is more likely to affect the alveolar bone whose level is closer to it. To be specific, the palatal alveolar bone resorption at the mid-root was correlated with the movement of the mid-point of the incisor neck, while the palatal alveolar bone resorption at the apical level with the root apex point; and although the labial alveolar bone resorption at the apical level was associated with all four points, the correlation coefficients increased with the point being closer (rR = 0.495 > rM = 0.485 > rL = 0.349 > rC = 0.280). However, this regularity was not observed in the maxillary central incisor. This could be explained by the fact that the movement types of the maxillary and mandibular central incisors are normally not the same in extraction cases with moderate anchorage. Maxillary central incisors tend to present movement between crown tipping and bodily movement because of the positive torque moment in the brackets, while mandibular central incisors present the tipping movement with little root movement [27,28].

There has been a consensus that during orthodontic tooth movement (OTM), alveolar bone remodeling is a balance between bone resorption and regeneration, and as shown in some previous studies, after incisor retraction, a rise in the thickness of the labial or palatal alveolar bone may occur [29,30,31,32]. Admittedly, in this study, the alveolar bone changes were all bone resorption, and no bone regeneration was observed, neither on the labial nor on the palatal side nor at any of the levels. However, this phenomenon could be explained from two aspects. The first aspect to consider is the temporal sequences of OTM. The mechanism of alveolar bone remodeling involves responding to the stimulation of orthodontic force: on the compression side, osteoclasts would appear, and the alveolar bone would be resorbed; while on the tension side, osteoblasts would appear, and the alveolar bone would regenerate [33]. However, cell activation and differentiation are not simultaneous; cathepsins and matrix metalloproteases (MMPs), the two enzymes that contribute to bone resorption, would increase on the compression side at the early stage of OTM [34]. Hence, alveolar bone formation tends to be slower than bone resorption. In this study, CBCT images were obtained within only two weeks after finishing treatment, which was a too short period of time for bone regeneration; therefore, alveolar bone regeneration was not observed in all the data. The other aspect pertains to the average alveolar bone loss in adults. As has been revealed in previous studies, there is an overall rate of alveolar bone loss of about 0.02–0.09 mm per year in general populations [35,36]. Alveolar bone loss is closely related to smoking, age, gender, etc. [37,38]. Even in patients taking orthodontic treatment without premolar extraction, alveolar bone loss was also observed [39].

This study has several advantages. Firstly, it innovatively uses four landmarks to describe the movement of the incisor, which help better understand its three-dimensional movement. Secondly, compared with other similar studies that include several tens of samples, our sample size is considerably larger, which potentially increases the reliability of the results. Thirdly, because the method we used for data preparation in this study has a stable maxillary-based coordinate system and mandibular voxel-based superimposition, which eliminates the interference of mandibular position change, we could accurately locate the landmarks and conduct three-dimensional measurements.

This study has a few limitations. One limitation was the sex distribution in the samples. Only ten males were selected, probably due to the disparate willingness between adult males and females to receive orthodontic treatment [40]. Secondly, as mentioned, the alveolar bone level was evaluated within a very short period of time after finishing treatment; thus, further studies are still needed to understand the alveolar bone changes after periodontal reconstruction and stabilization. Thirdly, as the voxel size we used for CBCT images in this study was 125 μm, according to previous studies, linear measurements might show more or less overestimation or underestimation [41]. Hence, we remind readers that potential measurement errors may be encountered with CBCT, which should be taken into consideration.

Our findings revealed a relatively regular and concrete pattern of how alveolar bone changes follow the incisor retraction in adults with premolar extraction. This is clinically meaningful for orthodontic treatment. On the one hand, it is instrumental for orthodontists to have a relatively accurate prediction of alveolar bone resorption based on the specific movements of central incisors. On the other hand, it may help orthodontists to reduce the risk of undesirable alveolar bone resorption via better analyzing and adjusting the three-dimensional movement types of incisors.

## 5. Conclusions

This study systematically investigated, in a detailed and comprehensive way, the correlation between the movement of maxillary and mandibular central incisors and alveolar bone resorption in adults who had orthodontic premolar extraction treatment. By providing a more concrete understanding of their inter-correlation, on the one hand, this study could potentially be helpful for orthodontists to have a relatively accurate prediction of alveolar bone resorption based on the specific movements of central incisors, while on the other, it could assist orthodontists to better adjust the three-dimensional movement types of incisors to avoid undesirable alveolar bone resorption.

## Figures and Tables

**Table 1:** Dental landmarks for measuring incisor movement.

Dental Landmarks	Definition
C	The cusp point of the incisor
R	The root apex point of the incisor
L	The cementoenamel junction (CEJ) point closest to the labial side
M	The mid-point of the line between point L and the CEJ point closest to the lingual side

The definition and location of the dental landmarks were based on previous research studies [21].

**Table 2:** Planes for evaluating the angular changes in incisors and alveolar ridge.

Planes	Definition
PP	The palatal plane, the horizontal plane of the coordinate system
MP	The mandibular plane, constructed by Point Gn and the bilateral Point Go
Li	The long axis of the incisor, the line passing through Point C and Point R
U1	The labial alveolar ridge measurement plane, the tangent plane through the apex point to the labial alveolar ridge
U2	The palatal alveolar ridge measurement plane, the tangent plane through the apex point to the palatal alveolar ridge

The definition and location of the dental landmarks were based on previous research studies [10,22].

**Table 3:** Measurement landmarks for evaluating the alveolar bone thickness at crestal, mid-root, and apical levels.

Measurement Landmarks	Definition
A1	The apex point of the labial alveolar ridge
A2	The labial alveolar ridge point at the mid-root level
A3	The labial alveolar ridge point at the apical level
B1	The apex point of the palatal alveolar ridge
B2	The palatal alveolar ridge point at the mid-root level
B3	The palatal alveolar ridge point at the apical level

The definition and location of the alveolar bone landmarks were based on previous research studies [23,24].

**Table 4:** Baseline characteristics of subjects.

Patients (n)	63
Central incisors (n)	252
Maxillary central incisors (n)	126
Mandibular central incisors (n)	126
Initial age (y)	Mean 24.41 SD 5.80
Sex	
Male	10 (15.87%)
Female	53 (84.13%)
Treatment duration (months)	Mean 31.77 SD 10.30

**Table 5:** Difference between the two sexes in terms of age and treatment duration.

Characteristic	Male	Female	*p*-Value
Patient numbers	10	53	
Age (year)	24.30 ± 5.75	24.30 ± 4.80	0.699
Treatment duration (month)	31.25 ± 16.00	32.00 ± 15.50	0.721

**Table 6:** Movement of the dental landmarks of maxillary and mandibular central incisors.

	Mean (mm)	SD
Maxillary		
Point C	3.86	2.13
Point R	2.43	1.32
Point L	2.86	1.54
Point M	2.83	1.53
Mandibular		
Point C	4.34	2.17
Point R	3.25	1.81
Point L	3.69	1.85
Point M	3.67	1.83

**Table 7:** The thickness changes in the maxillary alveolar bone at different levels.

	Pre-Treatment		Post-Treatment		*p*-Value
	Mean (mm)	SD	Mean (mm)	SD	
A1–Li	1.38	0.76	1.13	0.74	0.001 **
A2–Li	1.71	0.74	1.39	0.77	0.000 ***
A3–Li	3.24	1.36	2.75	1.10	0.012 *
B1–Li	1.42	0.69	1.41	0.82	0.887
B2–Li	2.91	1.50	2.56	1.58	0.003 **
B3–Li	7.12	1.76	6.74	2.13	0.054

* *p* < 0.05; ** *p* < 0.01; *** *p* < 0.001.

**Table 8:** The thickness changes in the mandibular alveolar bone at different levels.

	Pre-Treatment		Post-Treatment		*p*-Value
	Mean (mm)	SD	Mean (mm)	SD	
A1–Li	1.04	0.74	1.05	0.67	0.756
A2–Li	1.06	0.75	1.09	0.67	0.499
A3–Li	3.70	1.64	2.92	1.13	0.000 ***
B1–Li	1.01	0.56	0.94	0.54	0.169
B2–Li	1.65	0.90	1.28	0.85	0.000 ***
B3–Li	4.46	1.17	3.63	1.47	0.000 ***

*** *p* < 0.001.

**Table 9:** The inclination changes in the central incisor and its associated labial and palatal alveolar ridge.

	Pre-Treatment		Post-Treatment		*p*-Value
	Mean (°)	SD	Mean (°)	SD	
Maxillary					
Li–PP	94.06	29.46	93.15	25.87	0.546
U1–PP	90.77	36.96	89.29	33.05	0.248
U2–PP	97.60	33.65	95.69	30.55	0.337
Mandibular					
Li–MP	100.39	28.06	103.31	24.02	0.118
U1–MP	102.73	30.17	106.04	25.92	0.091
U2–MP	95.87	27.27	97.36	22.54	0.513

**Table 10:** Factors correlated with maxillary alveolar bone resorption using Spearman correlation analysis.

	Age	Treatment Duration	Point C	Point R	Point L	Point M
A1–Li	0.215	0.015	−0.045	−0.087	−0.362 **	0.066
A2–Li	−0.015	0.176	−0.138	0.138	−0.187	−0.041
A3–Li	0.177	−0.136	0.254 *	0.225	0.196	0.387 **
B2–Li	−0.020	0.034	0.287 *	−0.088	0.098	0.125

* *p* < 0.05; ** *p* < 0.01. Significant correlations below the Bonferroni cut-off (*p* < 0.0021) are in bold.

**Table 11:** Factors correlated with mandibular alveolar bone resorption using Spearman correlation analysis.

	Age	Treatment Duration	Point C	Point R	Point L	Point M
A3–Li	−0.046	0.056	0.280 *	0.495 ***	0.349**	0.485 ***
B2–Li	−0.158	0.043	0.102	0.091	0.144	0.296 *
B3–Li	−0.011	0.207	0.158	0.354 **	0.188	0.247

* *p* < 0.05; ** *p* < 0.01; *** *p* < 0.001. Significant correlations below the Bonferroni cut-off (*p* < 0.0028) are in bold.

**Table 12:** Correlations between maxillary central incisor movement and alveolar bone resorption using multiple linear regression analysis.

	Point C	Point L	Point M
A1–Li		0.290 *	
A3–Li	−0.108		0.387 *
B2–Li	−0.170		

* *p* < 0.05.

**Table 13:** Correlations between mandibular central incisor movement and alveolar bone resorption using multiple linear regression analysis.

	Point C	Point R	Point L	Point M
A3–Li	−0.288	0.498 **	−0.142	−0.493 *
B2–Li			-	−0.170 **
B3–Li		0.177 **		

* *p* < 0.05; ** *p* < 0.01. Significant correlations below the Bonferroni cut-off (*p* < 0.0083) are in bold.

## Data Availability

Data can be provided upon reasonable request from the corresponding author.
